# Stress Measured by Allostatic Load Varies by Reason for Immigration, Age at Immigration, and Number of Children: The Africans in America Study

**DOI:** 10.3390/ijerph17124533

**Published:** 2020-06-24

**Authors:** Thomas Hormenu, Elyssa M. Shoup, Nana H. Osei-Tutu, Arsene F. Hobabagabo, Christopher W. DuBose, Lilian S. Mabundo, Stephanie T. Chung, Margrethe F. Horlyck-Romanovsky, Anne E. Sumner

**Affiliations:** 1 Section on Ethnicity and Health, Diabetes, Endocrinology, and Obesity Branch, National Institute of Diabetes and Digestive and Kidney Diseases, Bethesda, MD 20892, USA; thormenu@ucc.edu.gh (T.H.); elyssa.shoup@nih.gov (E.M.S.); nana.osei-tutu@nih.gov (N.H.O.-T.); hobabagabo@gmail.com (A.F.H.); christopher.dubose@nih.gov (C.W.D.); lilian.mabundo@nih.gov (L.S.M.); stephanie.chung@nih.gov (S.T.C.); 2 National Institute of Minority Health and Health Disparities, Bethesda, MD 20892, USA; 3 Department of Health and Nutrition Sciences, Brooklyn College, City University of New York, Brooklyn, NY 11210, USA; MargretheHR@brooklyn.cuny.edu

**Keywords:** allostatic load score, stress, African immigrants, cardiometabolic health

## Abstract

Stress leads to physiologic dysfunction and cardiometabolic disease. Allostatic load score (ALS) measures stress-induced cardiovascular, metabolic, and inflammatory biomarkers. We estimated the odds of high ALS by reason for and age at immigration, duration of American residence, number of children, and socioeconomic status in 193 African immigrants (male: 65%, age 41 ± 10 y (mean ± Standard Deviation (SD)), range 22–65 y). ALS was calculated with High-ALS defined as ALS ≥ 3.0 and Low-ALS defined as ALS < 3.0. Oral glucose tolerance tests (OGTT) were performed, the cardiovascular disease (CVD) risk estimated, and TNF-α, an inflammatory cytokine, measured. Logistic regression was used to estimate odds of High-ALS. In the High- and Low-ALS groups, ALS were 4.0 ± 1.2 vs. 1.3 ± 0.7, diabetes prevalence: 14% vs. 4%, CVD risk: 23% vs. 8%, TNF-α levels: 15 ± 9 vs. 11 ± 6 pg/mL, respectively (all *p* ≤ 0.01). Immigrants were more likely to be in the High-ALS group if their reason for immigration was work or asylum/refugee (OR 2.18, *p* = 0.013), their age at immigration was ≥30 y (OR 3.28, *p* < 0.001), their duration of residence in United States was ≥10 y (OR 3.16, *p* = 0.001), or their number of children was ≥3 (OR 2.67, *p* = 0.019). Education, income, health insurance, marital status, and gender did not affect High-ALS odds. Factors adversely influencing allostatic load and cardiometabolic health in African immigrants were age at and reason for immigration, duration of residence in America, and number of children.

## 1. Introduction

Cardiometabolic health is essential to a person’s well-being. Assessing the cardiometabolic health status of the African-born community living in the United States is challenging because of the rapid increase in the size of the community and the lack of appreciation of the unique stress faced by African immigrants [1,2,3,4]. The Migration Policy Institute reports that between 2010 and 2018 the African-born population increased by 52% compared to a 12% increase in the foreign-born population overall [4]. To provide temporal context, prior to 1980 less than 150,000 Africans resided in the United States. By 2018, the African community in the United States was estimated to be 2.1 million (4.8% of the foreign-born population) [5].

In the transition from life in sub-Saharan African countries to life in the United States, Africans must navigate new social norms, frequent underemployment, and changes in family structure [6]. Allostatic load is a way to understand the effect of these stressful changes on health. The allostatic load concept, pioneered by Bruce McEwen, is defined as stress-induced physiologic dysregulation due to chronic overactivation of the hypothalamic–pituitary–adrenal and sympathetic–adrenal–medullary axes [7,8]. Repeated exposure to stress leads to excess secretion of glucocorticoids and catecholamines and increases the risk for diabetes, cardiovascular disease (CVD), and inflammatory disorders [7]. Allostatic load score (ALS) is a direct measure of stress-induced biomarkers in three domains: cardiovascular, metabolic, and immune [7,8]. The higher the ALS, the greater the physiologic dysfunction.

Allostatic load has been studied in both African Americans and foreign-born blacks. However, with the exception of our previous research with the Africans in America cohort, we do not believe that ALS has been specifically studied in African immigrants [1,3,9,10,11]. The challenge is that most epidemiological surveys, including National Health and Nutrition Examination Survey (NHANES), combine all foreign-born blacks into a single group. As African descent populations are diverse in culture, history, and genetics, there are differences in allostatic load triggers, as well as the etiology and presenting phenotype of diabetes and CVD [12,13,14,15].

Despite the paucity of research on ALS in African immigrants, the prevalence of diabetes and heart disease has been examined [16,17,18]. However, these studies often rely on a single lipid profile for CVD risk assessment and on self-report, HbA_1c_, or fasting glucose for diabetes diagnosis [16,17,18]. These latter two tests lead to a significant under-diagnosis of diabetes in Africans [19,20]. In addition, self-report of diabetes in African immigrants may be especially inadequate because Africa has the highest percentage of people living with diabetes who are undiagnosed [21]. If Africans with undiagnosed diabetes emigrate and have limited access to medical care in the United States, they will remain undiagnosed. 

ALS analyses have some distinct advantages which extend beyond providing data on disease prevalence. ALS is a simultaneous assessment of physiologic dysfunction in three domains: cardiovascular, metabolic, and immune [9]. Furthermore, in the Africans in America cohort, we supplement our investigations of ALS with oral glucose tolerance tests (OGTT) for definitive diabetes diagnosis, measurement of five inflammatory markers not included in the ALS, and abdominal computerized tomographic scans for visceral adipose tissue (VAT) measurement. VAT is a key risk factor for diabetes and heart disease [22]. 

There are numerous ALS equations available [23]. For three reasons, we chose to use the 10 variable equation published by Chyu et al. [9]. First, there are sex-differences in many of the variables included in ALS equations (body mass index (BMI), blood pressure (BP), and high density lipoprotein (HDL)). Chyu et al. determined the threshold for risk for each equation variable by using sex-specific values. Second, the equation used by Chyu et al. determines risk thresholds for each variable by the population distribution of the group studied rather than by a value defined by a specific laboratory as normal. Normative values are often based on studies of white populations and their applicability to other populations is uncertain [24,25,26]. Third, as a cardiac marker of risk, the Chyu et al. equation uses high density lipoprotein (HDL) rather than triglyceride (TG). In populations of African descent, HDL is superior to TG as a marker of insulin resistance [3,24,25,27,28]. Furthermore, in our earlier research with the Africans in America cohort, we found that ALS equations which used HDL rather than TG to identify stress performed better [3]. 

With a focus on immigrants who came to the United States as adults (≥18 years of age), we determined the following in the Africans in America cohort: (a) the relationship of ALS to diabetes, CVD, and markers of inflammation; and (b) the odds of experiencing stress because of the reason for immigration, age at immigration, duration of residence in the United States, family responsibilities, and socioeconomic status.

## 2. Materials and Methods 

### 2.1. Population

The Africans in America cohort was designed to assess the cardiometabolic health of African-born blacks living in the United States [19,22,29]. Recruitment was achieved with the use of the NIH website, newspaper advertisements, flyers, and community event presentations. Study approval was obtained from the NIDDK Institutional Review Board (Clinical Trials.gov Identifier: NCT00001853). Each participant gave written informed consent.

Prior to enrollment, a telephone interview was conducted. Potential participants had to state that they were born in a sub-Saharan African country to two black parents who were also born in sub-Saharan Africa. In addition, they had to self-identify as healthy with no history of diabetes, hypoglycemic, or hypolipidemic medications.

Four hundred and forty-three African-born blacks living in the Washington, DC, area successfully completed the telephone interview and proceeded to Visit 1, which was an outpatient screening visit at the NIH Clinical Center, Bethesda, Maryland (Figure S1). Social and medical histories, a physical, an electrocardiogram, and routine blood tests were performed. After Visit 1, 32 individuals were excluded. The reasons were anemia, elevated liver transaminases, hypothyroidism, IV access issues, or scheduling challenges. 

Therefore, 411 enrollees proceeded to Visit 2 for measurement of weight, height, blood pressure (BP), waist circumference, and an OGTT (Trutol 75, Custom Laboratories). In addition, blood samples were obtained for HbA_1c_, hemoglobin electrophoresis, an inflammatory panel, and the other biomarkers used for the calculation of the ALS. Furthermore, at Visit 2, an abdominal computerized tomographic scan was performed on 186 individuals (Siemens and Somatom Force Scanner) to measure visceral adipose tissue (VAT). 

Two individuals were excluded after Visit 2 because hemoglobin electrophoresis revealed hemoglobin-type AF. Persistent fetal hemoglobin interferes with the determination of HbA_1c_ by high performance liquid chromatography (HPLC) [30]. As HbA_1c_ is one of the 10 biomarkers included in the ALS equation, these two individuals could not go forward. Hence, the sample size for the calculation of the population-based ALS was 409. 

After the ALS calculation was complete, 216 individuals were excluded. Of these 216 individuals, 135 consecutively enrolled individuals were not asked to provide a reason for immigration. The lack of this critical data point led to their exclusion. In addition, 6 of the 216 who were in the group asking for the reason of United States entry were ineligible. Their reason for United States entry was either vacation or medical treatment. Seventy-five of the 216 individuals not included in this analysis had immigrated to the United States as minors (<18 years). This investigation focuses on the 193 immigrants who came to the United States as adults (≥18 years).

### 2.2. Reasons for Immigration

‘Reason for immigration’ was asked as an open-ended question by a single investigator (AES). There were no follow-up questions, no documentation requested, and no one declined to answer the question. 

The reasons the participants for United States entry gave were work, asylum, refugee, study, family reunification, and diversity visa lottery. The two categories asylum (*n* = 30) and refugee (*n* = 9) were combined into a single group (asylum/refugee).

### 2.3. Determination of Allostatic Load Score

The calculation of ALS requires identification of high-risk thresholds for 10 biomarkers in three domains: cardiovascular (systolic BP, diastolic BP, pulse, cholesterol, high density lipoprotein (HDL), homocysteine), metabolic (BMI, HbA_1c_), and immune (high sensitivity C-reactive protein (hsCRP), albumin) [9]. To identify thresholds for the high-risk quartile for each biomarker, the cohort of 409 participants was divided into sex-specific quartiles. High-risk was defined as a value above the 75th percentile for all biomarkers except HDL and albumin for which the value below the 25th percentile was used. Then, the biomarkers were made dichotomous with 1 point given if the biomarker was in the high-risk range and 0 if not. Use of antihypertensive medication led to the assignment of the high-risk category for both systolic and diastolic BP. High-ALS and Low-ALS groups were empirically defined by the threshold at the upper third of the population distribution of ALS (≥3 and <3, respectively).

### 2.4. Diabetes Diagnosis

Diagnosis of diabetes was based on glucose criteria for the OGTT, specifically fasting glucose ≥126 mg/dL and/or 2 h glucose ≥200 mg/dL [30].

### 2.5. 10-Year Cardiovascular Disease Risk Estimate

The calculation of a 10-year CVD risk was estimated using the Pooled Cohort Equations (PCE) for black men and women [2]. The variables included in the PCE were sex, age, cholesterol, high density lipoprotein (HDL), systolic blood pressure (including antihypertensive treatment), current smoking status, and diabetes. 

### 2.6. Assays

Hemoglobin and hematocrit were measured in EDTA-anticoagulated whole blood using a Sysmex XE-5000. Glucose, cholesterol, triglyceride, HDL, homocysteine, albumin, creatinine, fibrinogen, ferritin, and hsCRP were measured in plasma (Roche Cobas 6000 analyzer, Roche Diagnostics, Indianapolis, IN, USA). HbA_1c_ values were determined by HPLC using a National Glycohemoglobin Standardization Program (NGSP)-certified instrument (D10) made by BioRad Laboratories (Hercules, CA, USA).

TNF-α and MCP-1 were measured in plasma using a Luminex bead array assay with the Multiplex kit (HCYTOMAG-60K-10, Millipore, Chicago, IL, USA) on a Bio plex 200 instrument using the Bio-plex 6.1 software (Bio-Rad, Hercules, CA, USA). Data were available for *n* = 168 participants. IL-6 was measured in plasma with a digital enzyme-linked immunosorbent assay (ELISA) using SiMOA technology (cat no. 101622, Quantrix, Billerica, MA, USA). IL-6 levels were available in *n* = 182.

### 2.7. Statistical Analyses

Unless stated otherwise, data are presented as mean ± SD. Analyses included unpaired *t*-tests, one-way analysis of variance (ANOVA) with Bonferroni correction for multiple comparisons, and chi-square tests. Logistic regressions were used for odds ratio estimates. *P*-values of ≤0.05 were considered significant. Data were managed with Research Electronic Data Capture (REDCap) [31]. Analyses were performed with STATA16 (College Station, Texas).

## 3. Results

The birth countries of the participants were divided in four areas of sub-Saharan Africa: West (49%), Central (15%), East (34%), and South (2%). The four participants who were from South African countries were analyzed with the Central African group. There was no difference by region in gender, age, age at immigration, duration of residence in the United States, or reason for immigration (Table S1). West Africans had the highest college graduation rate (*p* = 0.019). There was no difference by region in income or health insurance coverage. 

Heterozygous hemoglobinopathies (i.e., sickle cell trait and hemoglobin C trait) were more common in West and Central Africans than East Africans (*p* = 0.048).

There was no difference by region in body size, metabolic factors, prevalence of diabetes, 10-year CVD risk, or ALS (Table S1). Therefore, the participants from West, Central, and East Africa were combined into a single group. 

### 3.1. Gender Characteristics

There were many differences by gender in social characteristics and body size, but few metabolic differences (Table 1). Compared to men, women had a lower college graduation rate and were more likely to be unmarried. Women consumed less alcohol than men. None of the women smoked, but 9% of men did. Women reported a higher fruit and vegetable intake than men. Both groups were sedentary.

From a metabolic perspective, women were more obese, but had less VAT than men. However, the prevalence of diabetes, 10-year CVD risk, and ALS did not differ by gender. Fibrinogen concentrations were higher in women. Ferritin levels were higher in men. TNF-α, MCP-1, and IL-6 did not differ by gender.

### 3.2. ALS by Reason for Immigration

**Figure 1:**
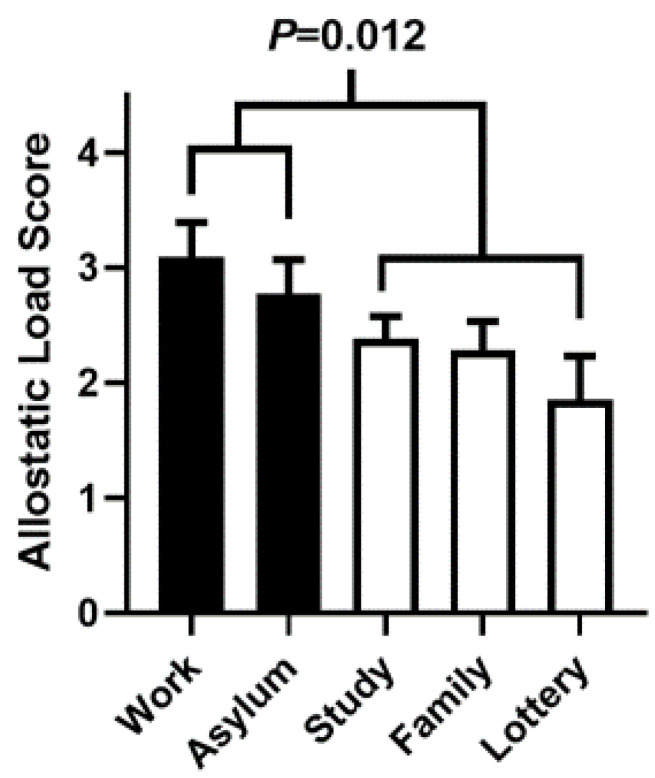
Allostatic Load Score by Reason of Immigration. Data presented as mean ± SE. *P*-value for downward trend across the 5 reasons of immigration is: 0.004. Mean ALS for [Work+Asylum/Refugee] vs. [Study+Family Reunification+Lottery] is: 2.93 + 1.64 vs. 2.30 + 1.63, *p* = 0.012.

From highest to lowest ALS, the reasons for immigration were work (3.10 ± 1.67), asylum/refugee (2.77 ± 1.65), study (2.38 ± 1.70), family reunification (2.38 ± 1.59), and diversity visa lottery (1.86 ± 1.41) (Figure 1). The *P*-value for the downward trend of ALS was significant (*p* = 0.004) (Figure 1). In addition, there was a significant difference in mean ALS when comparing [Work + Asylum/Refugee] to [Study + Family Reunification + Lottery] (2.93 ± 1.64 vs. 2.30 ± 1.63, *p* = 0.012) (Figure 1). This is our basis for identifying work and asylum/refugee as high-risk reasons for immigration. Study, family reunification, and diversity visa lottery are identified as low-risk reasons.

### 3.3. Characteristics by Group: High-ALS versus Low-ALS

Eight of the 10 ALS equation variables were higher in the High-ALS group (Table 2). The variables which did not differ by ALS group were cholesterol (*p* = 0.082) and albumin (*p* = 0.518) (Table 2). 

Social and biographical variables which were higher in the High-ALS group were age at immigration (33 ± 9 vs. 30 ± 9 y, *p* = 0.013), duration of residence in the United States (12 ± 10 vs. 8 ± 8 y, *p* = 0.003), reason for immigration ([Work + Asylum/Refugee] vs. [Study + Family Reunification + Lottery]) (Figure 1), and having three or more children (36% vs. 22%, *p* = 0.031) (Table 2). 

All three measures of body size, BMI, WC, and VAT were higher in the High-ALS group than in the Low-ALS group (Table 2). In addition, the prevalence of diabetes and 10-year CVD risk was higher in the High-ALS group. Four out of the five inflammatory markers (fibrinogen, TNF-α, ferritin, and MCP-1) measured were higher in the High-ALS group. With a *P*-value of 0.197, IL-6 did not differ by ALS group.

### 3.4. Odds of Being in the High-ALS Group

To examine immigration-related factors which contributed to the odds of being in the High-ALS group, four models were created (Table 3). In all four models, gender was included and did not influence the odds of being in the High-ALS group.

Model 1 included age at immigration and duration of residence in the United States. African-born blacks were three times more likely to be in the High-ALS group if the duration of residence in the United States was ≥10 y (OR 3.16, *p* = 0.001). Sensitivity analyses of immigration at ≥30, ≥40, and ≥50 years revealed that an immigration age of ≥30 y had three times the odds of being in the High-ALS group (3.28, *p* < 0.001) (Table S2). Immigration after ages 40 or 50 did not increase the odds further.

Model 2 was based on the finding that ALS was higher if reasons for immigration were [Work + Asylum/Refugee] rather than [Study + Family + Lottery] (4.0 ± 1.2 vs. 2.3 vs. 0.7, *P* < 0.001) (Figure 1). The odds of being in the High-ALS group were twice as high if reasons for immigration were [Work + Asylum/Refugee] than if reasons for immigration were [Study + Family + Lottery] (OR 2.17, *P* = 0.013).

Model 3 focused on family responsibilities and included the number of children and marital status. Marital status did not modify stress category. Sensitivity analysis established that a number of children of ≥3 was the appropriate cut-point for ALS odds (OR: 2.67, *P* = 0.019). Odds of being in the High-ALS group did not reach significance if there were only one or two children in the family (Table S3).

Model 4 included factors related to socioeconomic status, specifically income, education, and health insurance. As all three odds ratios were ~1.0, the odds of being in the High-ALS group were not modified by any of these factors.

## 4. Discussion

The Africans in America cohort brings new insight to the relationship between allostatic load, immigration-induced stress, and risk for cardiometabolic disease. In addition, our research with ‘duration of stay in the United States’ confirms well-known observations about the immigration experience. Overall, we found that African immigrants had higher odds of being in the High-ALS group (a) if their reason for immigration was work or asylum/refugee, (b) if immigration was undertaken after the age of 30 years, and (c) if they had three or more children. Consistent with other reports, we also found that African immigrants had higher odds of being in the High-ALS group if their duration of residence in the United States was 10 years or greater [32]. In addition, higher education, higher income, and health insurance did not appear to lower allostatic load and offset the stress of immigration [11].

### 4.1. Allostatic Load Score

ALS reflects the influence of chronic stress on cardiometabolic health. Indeed, we found that both CVD risk and diabetes prevalence were higher in the High-ALS than in the Low-ALS group (both *p* ≤ 0.01). Concern about high ALS in African immigrants is clearly warranted. In the MacArthur studies of successful aging, higher ALS predicted a higher rate of all-cause mortality [33]. Furthermore, a prospective study of ALS conducted for over 10 years in Taiwan revealed that for every 1-point increase in ALS, mortality increased [34]. In our study, the difference in mean ALS between the High-ALS and Low-ALS groups was ~3 points.

Another reason for concern about High-ALS relates to inflammation. Emerging research suggests that inflammatory biomarkers may both predict incident diabetes and have an etiological role in the development of diabetes [35,36]. In the Africans in America cohort, four of five markers of inflammation (fibrinogen, TNF-α, ferritin, and MCP-1) were higher in the High-ALS group. The fifth one, IL-6, approached significance. As none of these inflammatory markers were included in the ALS equation, the high concentrations of these biomarkers in the High-ALS group illustrates the ability of ALS to identify risk.

### 4.2. Reason for Immigration

As we found significant differences in the ALS created by the mean ALS for [Work + Asylum/Refugee] versus the mean ALS for [Study + Family + Lottery], we identified work and asylum/refugee as high-risk reasons for immigration, and study, family reunification, and diversity visa lottery as low-risk reasons. This outcome represents an extension of our earlier research with the Africans in America cohort [3]. When the size of the adulthood immigrant cohort was fifty percent smaller, we found that ALS was lower when the reason for immigration focused on family unity [3]. Now that the cohort has doubled in size, it was possible to confirm that ALS is highest in the individuals who came to the United States for either work or asylum/refugee. In short, there are physiologic consequences for having to immigrate for these reasons. 

### 4.3. Duration of Residence 

For African immigrants, the longer the duration of residence in their destination country, the greater the stress, the higher the ALS, and the greater the deterioration of cardiac health [37]. This finding has been documented in African immigrants that migrated to the United States, as well as Ghanaians who have migrated to European countries [2]. Interestingly, the same observation has been made in Mexican immigrants that migrated to the United States [32]. 

Therefore, it is not surprising that in the Africans in America cohort, immigrants who had lived in the United States for more than 10 years had three times the odds of being in the High-ALS group than immigrants who had been in the United States for less than 10 years. A ten-year residence in the destination country is generally accepted as a marker of acculturation [32,37].

Reasons as to why health deteriorates with an increased duration of stay in the United States (or Europe) are uncertain. A major postulate is the adoption of unhealthy assimilation behaviors, such as increase in alcohol intake and a decrease in physical activity [2]. We did find that both alcohol intake and a sedentary lifestyle tended to be higher in the High-ALS group. However, as this is a cross-sectional study, we cannot gauge how these behaviors might have changed over time. 

We did not note a difference in smoking between the High- and Low-ALS groups. However, only 9% of African men and none of the African women in the cohort reported that they were smokers. As smoking is not characteristically a part of African culture, smoking is a suboptimal marker of unhealthy assimilation behavior in this population. 

Another factor which can lead to deteriorating health in immigrants is depression [2]. While we do not have mental health data, we speculate that with increased duration of stay in the United States, there could be a growing realization that in America parental authority is not traditionally respected and a return to their country of origin is progressively less feasible [38].

### 4.4. Age of Immigration

As a continuous variable, higher age of immigration is associated with greater physiologic stress, higher ALS, and presumably worse cardiometabolic health [1,32]. We are not aware of studies in African immigrants which have tried to identify a threshold for age at immigration at which stress increases substantially. To provide a clinical context to the concept that a higher age during immigration has an adverse effect on ALS, we tested thresholds ages of 30 y, 40 y, and 50 y and found that with immigration above the age of 30, ALS increased substantially (Table 3 and Table S2). This finding speaks to the resilience and adaptability associated with young adulthood at an age of <30 y and the fact that the age at immigration is a biographical data point with important health implications.

### 4.5. Gender and Family Responsibilities 

There is great diversity genetically, geographically, and culturally in African descent populations [15,39]. This may explain the differences we are observing in reports by gender in ALS and CVD risk in African descent populations. For example, Geronimus et al. analyzed data from NHANES IV, 1999–2002 and reported that the ALS load was higher in African American women than men [11]. In contrast, we found no difference by gender in ALS in the Africans in America cohort. Investigators studying Ghanaian immigrants living in Europe did not measure ALS but did examine the CVD risk score [2]. While we found no difference in CVD risk between African men and women in the United States, they found lower CVD risk in Ghanaian women than men who were living in Europe [2]. These three different gender patterns in African Americans, Africans in the United States, and Africans in Europe may reflect differences in lifestyles, social pressures, and responsibilities.

Overall, influence of family and family responsibilities are difficult to assess. Easily measurable factors include the marital status and the number of children. As reasons for immigration which focused on family (family reunification and diversity visa lottery for oneself and immediate family) were associated with lower ALS, we had expected to find that marriage would have led to a decreased ALS. After all, marriage expands links between families. However, we found that marriage had no influence on ALS. Interestingly, Doamekpor et al. reported in foreign-born blacks enrolled in NHANES 2001–2010 that married individuals had higher ALS than individuals who never married [10].

The number of children is another accessible and measurable factor. We found that having three or more children was associated with more than twice the odds of being in the High-ALS group than having either no children or just one or two. In the United States, supporting children is expensive. In addition, Americanized children may not respond to parental authority in the same way that children living in Africa do. This could create stress for African parents of Americanized children [38]. 

### 4.6. Education and Income

A college education, income, and health insurance did not influence the ALS group classification (Table 3, Model 4). For African Americans, the situation is similar. In a study contrasting ALS in black and white Americans, Geronimus et al. reported that ALS did not decline in black Americans as markers of socioeconomic status increased [11]. In contrast, for white Americans, Geronimus et al. reported that ALS declined with higher education and income [11]. This black/white difference in the United States has been attributed to racism and perceived discrimination and could increase allostatic load in African immigrants. However, for African immigrants there may be additional factors which explain why education and income did not mitigate stress and lower ALS. If graduate degrees were obtained outside of the United States, whether awarded by African, European, Australian, or Asian universities, they may not be recognized in the United States. Lack of transferability of credentials leads to underemployment and an incongruence between education and income [6]. Furthermore, income earned in the United States may not lead to financial security because immigrants often send a significant portion of their income to relatives in their country of origin [4].

### 4.7. Strengths and Weaknesses

The greatest weaknesses of the study are the cross-sectional design, the size of the cohort, and the recruitment of a convenience sample via flyers and newspaper advertisements. Therefore, our sample could reflect bias via self-selection. However, immigration is a heterogeneous process with great diversity especially in regards to the area of United States residence. Nonetheless, our recruitment area included Washington, DC, which is described by the Migration Policy Institute as a top metropolitan destination for African immigrants [4]. Furthermore, two of the five counties with the highest concentrations of African-born blacks in the United States were in our recruitment area, Montgomery County, Maryland, and Prince Georges County, Maryland [4]. Plus, the greatest number of our participants are from West Africa. This is consistent with known migration patterns [4]. Furthermore, the sample size was large enough and sufficiently representative to detect known genetic differences by region. Specifically, sickle cell traits and hemoglobin C traits were significantly more common in participants from West and Central Africa than East Africa. Moreover, while the United States does not collect information on diabetes prevalence in immigrants according to country or region of birth, Canada does. According to the Ontario Diabetes Database, the prevalence of diabetes in sub-Saharan Africans living in Canada is 9% [40]. This is very similar to the 8% rate we discovered in the Africans in America cohort. 

## 5. Conclusions

ALS is a key link between stress and adverse health. Immigrants from Africa to the United States must deal with the stress of changing continents, changing cultures, and adjusting to life in America as a minority. Based on our research with African-born blacks living in the United States, physiologic stress is magnified when the reasons for immigration are work or asylum/refugee and immigration occurs after the age of 30. Additional challenges include coping with family responsibilities and providing for children. Overall, the adverse health consequences associated with immigration increase with the duration of stay in the United States and are not offset by education, income, or health insurance. To optimize health care and to decrease the risk for diabetes and CVD, medical providers should be sensitive to the interaction between stress and health in immigrants.

## Figures and Tables

**Table 1:** Participant characteristics by gender.

Parameters ^1^	Total *n* = 193 100%	Men *n* = 125 65%	Women *n* = 68 35%	*p*-Value ^2^
Age (y)	41 ± 10	40 ± 10	42 ± 10	0.154
Age at Immigration ≥30 years	50%	49%	53%	0.583
Immigration Reason: High-Stress ^3^	32%	32%	32%	0.960
United States Residence ≥10 years	42%	38%	50%	0.095
Married	56%	64%	41%	0.002
Number of Children ≥3 ^4^	28%	27%	31%	0.591
College Graduate	79%	83%	71%	0.041
Income ≥40k	46%	44%	50%	0.424
Health Insurance	68%	66%	74%	0.258
Current Smoker	6%	9%	0%	0.012
Alcohol ≥1 drink/week	21%	26%	13%	0.045
Total Daily Fruit/Veg ≥5 servings	13%	9%	20%	0.039
Sedentary Lifestyle ^5^	78%	81%	72%	0.132
BMI (kg/m^2^)	27.6 ± 4.2	26.6 ± 3.6	29.3 ± 4.7	<0.001
Waist Circumference (cm)	91 ± 11	91 ± 10	92 ± 13	0.407
VAT (cm^2^)	103 ± 66	115 ± 70	80 ± 53	0.001
Diabetes	8%	10%	4%	0.150
10-year CVD risk (%)	15 ± 17	14 ± 14	15 ± 23	0.715
Allostatic Load Score	2.5 ± 1.7	2.5 ± 1.6	2.5 ± 1.8	0.799
Fibrinogen (mg/dL)	280.9 ± 59.9	261.4 ± 53.8	316.4 ± 54.1	<0.001
TNF-α (pg/mL)	12.7 ± 7.2	13.0 ± 6.5	12.2 ± 8.5	0.505
Ferritin (µg/L)	136 ± 108	173 ± 108	67 ± 67	<0.001
MCP-1 (pg/mL)	282.2 ± 80.8	285.2 ± 82.3	276.7 ± 78.4	0.516
IL-6 (ng/mL)	2.6 ± 6.5	2.2 ± 2.5	3.6 ± 10.6	0.168

^1^ Data presented as mean ± SD or percentages; ^2^ comparison by unpaired *t*-test or Chi-square as appropriate; ^3^ high-stress reasons were work and asylum/refugee. Low-stress reasons were study, family reunification, and diversity lottery; ^4^ data available for *n* = 187; ^5^ International Physical Activity Category (IPAQ): low. BMI: body mass index; VAT: visceral adipose tissue; CVD: cardiovascular disease.

**Table 2:** Participant characteristics with High-allostatic load score (ALS) (ALS ≥ 3) and Low-ALS (ALS < 3).

Parameters ^1^	Total *n* = 193 100%	High-ALS *n* = 87 45%	Low-ALS *n* = 106 55%	*p*-Value ^2^
Allostatic Load Score	2.5 ± 1.7	4.0 ± 1.2	1.3 ± 0.7	<0.001
Systolic BP (mmHg)	119 ± 14	126 ± 15	113 ± 10	<0.001
Diastolic BP (mmHg)	72 ± 9	76 ± 9	69 ± 7	<0.001
Pulse (beat/min)	67 ± 10	69 ± 10	66 ± 9	0.038
Cholesterol (mg/dL)	167 ± 33	171 ± 35	163 ± 31	0.082
HDL (mg/dL)	54 ± 15	51 ± 16	56 ± 13	0.034
Homocysteine (mg/dL)	8.7 ± 3.2	9.4 ± 3.5	8.2 ± 2.8	0.007
BMI (kg/m^2^)	27.6 ± 4.2	29.5 ± 3.9	26.0 ± 3.7	<0.001
A1C (%)	5.4 ± 0.7	5.6 ± 0.8	5.2 ± 0.5	<0.001
Albumin (mg/L)	4.1 ± 0.2	4.1 ± 0.3	4.1 ± 0.2	0.518
hsCRP (mg/L)	1.8 ± 2.7	2.7 ± 3.7	1.1 ± 1.1	<0.001
Male	65%	62%	67%	0.477
Age (y)	41 ± 10	44 ± 10	38 ± 9	<0.001
Age at Immigration ≥30 years	50%	61%	42%	0.007
Immigration Reason: High-Stress ^3^	32%	41%	25%	0.013
United States Residence ≥10 years	42%	52%	34%	0.013
Married	56%	55%	57%	0.842
Number of Children ≥3 ^4^	28%	36%	22%	0.031
College Graduate	79%	78%	79%	0.855
Income ≥40k	46%	47%	45%	0.798
Health Insurance	68%	69%	68%	0.877
Current Smoker	6%	7%	5%	0.516
Alcohol ≥1 drink/week	21%	26%	17%	0.110
Sedentary Lifestyle ^5^	78%	75%	81%	0.298
BMI (kg/m^2^)	27.6 ± 4.2	29.5 ± 3.9	26.0 ± 3.7	<0.001
Waist Circumference (cm)	91 ± 11	97 ± 11	87 ± 10	<0.001
VAT (cm^2^)	103 ± 66	129 ± 68	83 ± 58	<0.001
Diabetes	8%	14%	4%	0.012
10-year CVD risk (%)	15 ± 17	23 ± 22	8 ± 7	<0.001
Fibrinogen (mg/dL)	280.9 ± 59.9	298.6 ± 63.5	265.8 ± 52.4	<0.001
TNF-α (pg/mL)	12.7 ± 7.2	14.8 ± 8.7	11.2 ± 5.6	0.001
Ferritin (µg/L)	136 ± 108	156 ± 108	118 ± 90	0.015
MCP-1 (pg/mL)	282.2 ± 80.8	299.2 ± 92.6	270.5 ± 69.5	0.023
IL-6 (ng/mL)	2.6 ± 6.5	3.4 ± 9.5	2.1 ± 2.5	0.197

^1^ Data presented as mean ± SD or percentages; ^2^ comparison by unpaired *t*-test or Chi-square as appropriate; ^3^ High-ALS reasons were work and asylum/refugee. Low-ALS reasons were study, family reunification, and diversity visa lottery; ^4^ data available for n = 187; ^5^ IPAQ Category: low.

**Table 3:** Odds of being in the High-ALS group (Logistic Regression).

Logistic Regression Models	Odds Ratio	95% CI	*p*-Value
Model 1: Immigration Age and Duration			
Age at Immigration ≥30 y vs. Age at Immigration <30 y	3.28	1.69, 6.36	<0.001
US Residence ≥10 y vs. US Residence <10 y	3.16	1.61, 6.19	0.001
Women vs. Men	1.05	0.56, 1.96	0.877
Model 2: Reason for Immigration			
[Work + Asylum/Refugee] vs. [Study + Family + Lottery]	2.17	1.18, 4.02	0.013
Women vs. Men	1.25	0.68, 2.27	0.476
Model 3: Family Responsibilities			
Children ≥3 vs. No Children	2.67	1.17, 6.09	0.019
Married vs. Not Married	0.81	0.34, 1.88	0.621
Women vs. Men	1.55	0.67, 3.57	0.303
Model 4: Socioeconomic Status			
Income ≥40K vs. Income <40K	1.07	0.58, 1.97	0.838
Health Insurance vs. No Health Insurance	1.01	0.52, 1.95	0.978
Education ≥College Degree vs. <College Degree	0.96	0.47, 1.95	0.912
Women vs. Men	1.23	0.67, 2.24	0.504

## Supplementary Materials

The following are available online at https://www.mdpi.com/1660-4601/17/12/4533/s1, Figure S1: Flow Diagram, Africans in America Cohort, Table S1: Participant Characteristics by African Region of Origin, Table S2: Odds of Being in the High-ALS Group by Age at Immigration (Logistic Regression), Table S3: Odds of Being in the High-ALS Group by Number of Children (Logistic Regression).
