# Supplementary material for: Stress Measured by Allostatic Load Varies by Reason for Immigration, Age at Immigration, and Number of Children: The Africans in America Study
Source: Int J Environ Res Public Health. 2020 Jun 24;17(12):4533. doi: 10.3390/ijerph17124533 (PMC7345091; doi:10.3390/ijerph17124533)
Supplement: Supplementary file 1 [file ijerph-17-04533-s001.pdf]

Supplement Figure 1

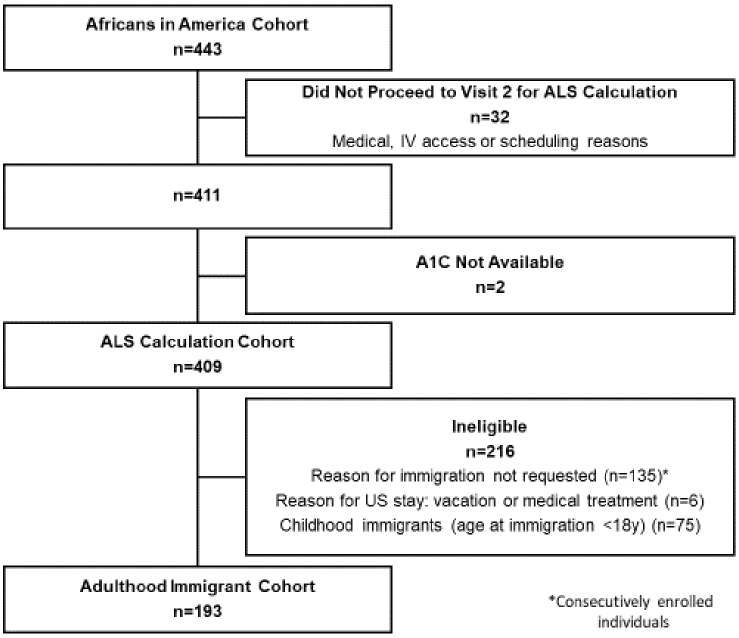

Figure S1. Flow Diagram, Africans in America Cohort.

Table S1: Participant Characteristics by African Region of Origin.

Parameter[1]	Total n = 193 100%	West n = 94 49%	Central n = 33 17%	East n = 66 34%	*p*-value[2]
**Male**	65%	68%	70%	58%	0.317
**Age (y)**	41±10	41±10	41±12	41±10	0.942
**Age at Immigration (y)**	31±9	30±8	31±9	32±9	0.213
**United States Residence (y)**	10±9	11±10	10±9	8±8	0.176
**Immigration Reason: High-Stress[3]**	32%	28%	45%	32%	0.169
**Number of Children ≥3[4]**	28%	24%	29%	34%	0.416
**Married**	56%	53%	48%	64%	0.270
**College Graduate**	79%	87%	70%	71%	0.019
**Income ≥40k**	46%	51%	30%	47%	0.118
**Health Insurance**	68%	70%	64%	68%	0.782
**Hemoglobin (g/dL)**	14.0±1.5	13.9±1.5	14.0±1.3	14.2±1.5	0.610
**Hematocrit (%)**	42.0±4.1	42.0±4.2	41.3±4.4	42.4±3.9	0.472
**Sickle Cell Trait**	15%	15%	24%	9%	0.048
**Hemoglobin C Trait**	3%	5%	0%	0%	0.048
**BMI (kg/m^2)**	27.6±4.2	28.1±4.1	27.0±4.4	27.1±4.2	0.241
**Waist Circumference (cm)**	91±11	92±11	88±11	91±12	0.232
**VAT (cm^3)**	103±66	102±63	102±78	104±67	0.989
**Diabetes**	8%	9%	6%	9%	0.870
**10-year CVD risk (%)**	15±17	15±18	18±19	13±16	0.380
**Allostatic Load Score**	2.50±1.66	2.62±1.75	2.33±1.73	2.42±1.51	0.629

[1] Data presented as mean±SD or percentages; [2] Comparison by One-Way ANOVA with Bonferroni correction or Chi-square as appropriate; [3] High-Stress reasons were: Work and Asylum-Refugee. Low-Stress reasons were: Study, Family Reunification and Diversity Lottery; [4] Data available for n = 187.

Table S2: Odds of Being in the High-ALS Group by Age at Immigration (Logistic Regression).

	Odds Ratio	95% CI	*p*-Value
**Model 1A: Immigration Threshold 30 years & US Residence Threshold 10 years**			
Age at Immigration ≥30y vs. Age at Immigration <30y	3.28	1.69, 6.36	<0.001
US Residence ≥10y vs. US Residence <10y	3.16	1.61, 6.19	0.001
Women vs. Men	1.05	0.56, 1.96	0.877
**Model 1B: Immigration Threshold 30 years**			
Age at Immigration ≥30y vs. Age at Immigration <30y	2.18	1.22, 3.90	<0.001
Women vs. Men	1.21	0.66, 2.21	0.538
**Model 2A: Immigration Threshold 40 years & US Residence Threshold 10 years**			
Age at Immigration ≥40y vs. Age at Immigration <40y	1.29	0.58, 2.87	0.540
US Residence ≥10y vs. US Residence <10y	2.12	1.16, 3.84	0.013
Women vs. Men	1.14	0.62, 2.09	0.674
**Model 2B: Immigration Threshold 40 years**			
Age at Immigration ≥40y vs. Age at Immigration <40y	1.08	0.50, 2.37	0.839
Women vs. Men	1.24	0.69, 2.25	0.475
**Model 3A: Immigration Threshold 50 years & US Residence Threshold 10 years**			
Age at Immigration ≥50y vs. Age at Immigration <50y	1.16	1.29, 4.54	0.836
US Residence ≥10y vs. US Residence <10y	2.06	1.15, 3.72	0.016
Women vs. Men	1.14	0.62, 2.10	0.666
**Model 3B: Immigration Threshold 50 years**			
Age at Immigration ≥50y vs. Age at Immigration <50y	1.00	0.26, 3.87	0.997
Women vs. Men	1.24	0.68, 2.25	0.478

Table S3: Odds of Being in the High-ALS Group by Number of Children (Logistic Regression).

	Odds Ratio	95% CI	*P*-Value
**Model 1: Family Responsibilities**			
Children ≥1 vs. No Children	1.95	1.00, 3.80	0.050
Married vs. Not Married	0.83	0.44, 1.56	0.562
Women vs. Men	1.24	0.66, 2.34	0.498
**Model 2A: Family Responsibilities**			
Children ≥2 vs. No Children	1.91	0.94, 3.91	0.075
Married vs. Not Married	0.81	0.40, 1.62	0.542
Women vs. Men	1.47	0.74, 2.89	0.279
**Model 3A: Family Responsibilities**			
Children ≥3 vs. No Children	2.67	1.17, 6.09	0.019
Married vs. Not Married	0.81	0.34, 1.88	0.621
Women vs. Men	1.55	0.67, 3.57	0.303
